# Tea Polyphenols Inhibit Methanogenesis and Improve Rumen Epithelial Transport in Dairy Cows

**DOI:** 10.3390/ani14172569

**Published:** 2024-09-04

**Authors:** Zhanwei Teng, Shuai Liu, Lijie Zhang, Liyang Zhang, Shenhe Liu, Tong Fu, Ningning Zhang, Tengyun Gao

**Affiliations:** 1 College of Animal Science and Veterinary Medicine, Henan Institute of Science and Technology, Xinxiang 453003, China; tengzhanwei@hist.edu.cn (Z.T.);; 2 College of Animal Science and Technology, Henan Agricultural University, Zhengzhou 450046, China; 3 Postdoctoral Research Base, Henan Institute of Science and Technology, Xinxiang 453003, China

**Keywords:** tea polyphenols, dairy cattle, rumen microbiome, methane emissions, rumen epithelial cell transport

## Abstract

**Simple Summary:**

The methane (CH_4_) production in ruminants not only decreases the efficiency of dietary energy utilization but also contributes to the greenhouse effect. The rumen epithelium serves as the primary site for the absorption of essential nutrients. This study systematically investigated the effects of tea polyphenols on methane production and the rumen epithelial cell transport capability in cattle using both in vitro and animal experiments, employing multi-omics techniques. In vitro results demonstrated that, compared to the control group, tea polyphenols significantly reduced CH_4_ production and the acetate/propionate ratio. Tea polyphenols reduce the generation of CH_4_ in the rumen by influencing the diversity and community structure of microbes such as bacteria, protozoa, and methanogens, thereby minimizing the loss of feed energy. Animal experiments showed that tea polyphenols significantly increased the concentrations of T-AOC and GSH-PX in bovine blood. In addition, by affecting the microbial community in the bovine rumen, tea polyphenols alter the fermentation patterns, thereby enhancing the host’s rumen epithelial cells’ capacity to transport VFAs. In production practice, the use of tea polyphenols can enhance the efficiency of nutrient utilization in dairy cows, thereby improving their productive performance.

**Abstract:**

This study systematically investigated the effects of tea polyphenols on methane (CH_4_) production and the rumen epithelial cell transport capability in cattle using both in vitro and animal experiments, employing multi-omics techniques. The in vitro results demonstrated that, compared to the control group, tea polyphenols significantly reduced CH_4_ production and the acetate/propionate ratio (*p* < 0.05). Tea polyphenols reduced CH_4_ production by inhibiting the relative abundance of *unclassified_d_Archaea* methanogens and the protozoa *Pseudoentodinium* and *g__Balantioides*. The animal experiments showed that tea polyphenols significantly increased the concentrations of T-AOC and GSH-PX in bovine blood (*p* < 0.05). In addition, microbial groups such as *Rikenellaceae_RC9_gut_group*, *Ruminococcaceae_NK4A214_group*, and *Butyrivibrio_2* were significantly enriched in the ruminal fluid of the tea polyphenol group (*p <* 0.05). The proteomic results indicated significant upregulation of proteins such as COIII, S100A8, FABP1, SLC2A8, and SLC29A1 (*p* < 0.05) and downregulation of proteins including HBB, RAB4A, RBP4, LOC107131172, HBA, and ZFYVE19 (*p* < 0.05), with FABP1 showing a positive correlation with propionate concentration, and RAB4A had a negative correlation (*p* < 0.05). Overall, tea polyphenols modulate the microbial composition within the rumen, inhibiting CH_4_ production and enhancing the host’s rumen epithelial cell transport capacity for volatile fatty acids.

## 1. Introduction

The methane (CH_4_) production in ruminants not only decreases the efficiency of dietary energy utilization but also contributes to the greenhouse effect [1]. CH_4_ losses from ruminant animals, accounting for 8% to 14% of their total energy intake, diminish the efficacy of energy utilization from feed. A reduction of 2.5 g in CH_4_ emissions per kilogram of standard milk leads to an increase of 300 mL of standard milk per kilogram of feed [2]. Therefore, controlling CH_4_ generation in the rumen is advantageous for enhancing the productivity of ruminant animals while mitigating adverse environmental effects. Approximately 85% of the CH_4_ emitted by ruminant animals originates from rumen fermentation, primarily through the conversion by the methanogenic bacteria of CO_2_, H_2_, methanol, formic acid, and other compounds into CH_4_ [3]. Furthermore, there exists a close symbiotic and interspecific hydrogen transfer relationship between rumen protozoa and methanogenic bacteria such that alterations in the number and community structure of rumen protozoa can result in changes in the population and arrangement of rumen methanogenic bacteria, thereby influencing CH_4_ production in the rumen [4].

The rumen is a crucial organ for the digestion and absorption of nutrients in ruminants. Rumen microbes ferment the ingested feed to produce nutrients such as volatile fatty acids (VFAs) and microbial crude protein, providing the nutrients necessary for the host’s growth, reproduction, and milk production, thus supporting various physiological activities [5,6]. The rumen epithelium serves as the primary site for the absorption of essential nutrients, such as VFAs, and acts as a specific hub for the interaction between the host and microbial metabolism [7]. VFAs contribute to more than 70% of ruminants’ digestive energy, with increased propionate levels linked to enhanced milk production [8,9]. 

Antibiotics have long been used for disease prevention, treatment, and promotion of animal growth [10]. In the context of restrictions or bans on antibiotic use in some countries (such as the European Union), plant polyphenols have garnered widespread attention among researchers due to their unique biological functions and “green” qualities. Studies have shown that plant polyphenols possess biological functions that alter rumen fermentation and maintain health [11], offering potential applications. Plant polyphenols have been shown to reduce CH_4_ emissions [12]. Studies have indicated variations in the modes of action of different plant extracts in the rumen [13]. Recent extensive studies in cattle and sheep have shown that incorporating appropriate amounts of plant secondary metabolites in diets can improve feed efficiency and enhance animal productivity [14]. However, research on the impact of plant secondary metabolites on rumen microbial communities remains sparse, and some mechanisms are still conjectural. The symbiotic relationship between the host and rumen microorganisms significantly influences CH_4_ production and animal productivity [15,16,17]. Investigating the mechanisms of animal energy acquisition and CH_4_ emission from a microbial community perspective is currently a key research area. Thus, the objective of the present study was to investigate the effects of tea polyphenols on CH_4_ production and rumen epithelium transport in cows through in vitro and animal experiments, employing 16S rDNA and proteomics techniques. The findings of this study offer valuable insights into the precise utilization of tea polyphenols in dairy cow production, contributing to the advancement of low-carbon, sustainable, and healthy practices within the dairy industry.

## 2. Materials and Methods

### 2.1. In Vitro Experimental Design and Experiment Materials

All animal experimental procedures were approved by the Animal Care and Use Committee of Henan Agricultural University (approval number: HENAU-2018-015). In vitro rumen fermentation experiment was designed as a single-factor trial for 5 treatments: control (CON, without polyphenols), tea polyphenols were added to the dry matter (DM) of the fermentation substrate at levels of 1%, 2%, 3%, and 4%, and each batch of fermentation included five blanks to correct for gas and CH_4_ production (the blank group only contained artificial rumen buffer, without substrate or polyphenols). Additionally, three independent incubation runs were performed. The in vitro culture lasted for 24 h, after which the volume of gas and CH_4_ production, as well as other rumen fermentation indicators, were measured.

For the in vitro experiments, peanut vine, sourced from the Henan Agricultural University Livestock Experiment Station, was selected as the experimental material. The peanut vine was air-dried (the chemical composition of peanut vine (DM basis) is CP 9.16%, NDF 49.03%, ADF 36.03%, crude fat 2.68%, crude ash 7.90%.), ground to pass through a 0.5 mm sieve, and stored in sample bags at room temperature for future use. The tea polyphenols used in this experiment were purchased from Xiya Reagents, with a grade of guaranteed reagent.

### 2.2. Artificial Saliva Composition

The rumen fluid utilized in this experiment was collected from three Holstein cows of similar age (3.5 to 4 years, body weight (560 ± 30 kg), non-lactation period, fitted with permanent ruminal cannulas at the Henan Agricultural University Livestock Experiment Station). Cows were housed in individual stalls, with free access to water. Diet was a total mixed ratio, fed twice daily. The detailed ingredient composition of the diet (dry matter (DM) basis) was 45% concentrate supplement, including gluten 10%, corn 34%, Soybean meal 15%, DDGS 15%, bran 15%, Cottonseed meal 5%, sesame meal 5%, premix 1% (Hefeng Feed Co., Ltd., Jiaozuo, China), 25% corn silage, 15% alfalfa hay, and 15% peanut vine. Two hours before the morning feeding on the day of this experiment, rumen fluid was sampled from various locations within the rumens of the three cannulated cows by syringe (150 mL). The collected rumen fluid was immediately transferred to a pre-warmed (39 °C) insulated lunch box filled with CO_2_ and quickly transported back to the laboratory. Prior to use, the rumen fluid was pooled and filtered through four layers of cheesecloth in an anaerobic chamber into a beaker, and the filtered fluid was maintained in a 39 °C water bath with continuous CO_2_ infusion to ensure an anaerobic environment.

The artificial saliva was prepared according to the method of Menke and Steingass [18], comprising five components as detailed in Supplementary Table S1 (S1). On the day of this experiment, artificial saliva was prepared according to the proportions shown in Supplementary Table S2 (S2), placed in a constant temperature water bath at 39 °C, and continuously bubbled with CO_2_ until the solution changed from light blue to colorless, at which point, it was used immediately.

According to the experimental design, one day before this experiment, accurately weighed 2 g of peanut vine powder was placed into labeled culture bottles and pre-warmed in a 39 °C incubator for 30–60 min before the start of this experiment on day two. The rumen fluid and artificial rumen buffer solution were then mixed in a 1:2 volume ratio, and 150 mL of this mixture was accurately dispensed into each culture bottle (CO_2_ was infused during this process to maintain an anaerobic environment). Immediately afterward, the stoppers and aluminum caps were secured tightly with a crimping tool and sealed with a film to ensure anaerobiosis. The bottles were then incubated in a 39 °C constant temperature incubator (Qingdao Haier Biomedical Co., Ltd., Qingdao, China) for 24 h for in vitro fermentation, during which they were shaken every two hours.

### 2.3. Measurement Indicators and Methods

#### 2.3.1. Total Gas Production Measurement

Fermentation was terminated at the 24th hour of each batch. The gas pressure inside the culture bottles was measured using a precision manometer (Shaanxi Meikong Electronic Technology Co., Ltd., Xi’an, China), and the total gas production (GP) for each experimental group was calculated using the following formula [19]:

GP = P × (V − 150)/(101.3 × W)

where GP is the actual gas production in the culture bottle (mL), P is the actual pressure reading from the manometer (Kpa), V is the volume of the fermentation bottle (mL), and W is the mass of the fermentation substrate (g).

#### 2.3.2. CH_4_ Production Measurement

CH_4_ content was collected using gas collection bags (Dalian Guangming Chemical Industry Gas Quality Monitoring Center Co., Ltd., Dalian, China) and analyzed using a gas chromatograph. The CH_4_ concentration was measured using a GC1120 gas chromatograph (Dongxi Analytical Instrument Co., Ltd., Beijing, China) equipped with a Flame Ionization Detector (FID). The analytical conditions were as follows: the column was a TP-porapak Q capillary column (15 m × 0.32 mm × 5 μm); the FID temperature was 200 °C; the injection port temperature was 150 °C; column temperature was 55 °C; the carrier gas was high-purity nitrogen with a flow rate of 10 mL/min; the standard sample concentration was 20.1 ppm with an injection volume of 0.3 mL. CH_4_ concentrations in the samples were calculated using external standardization based on the peak area of the standards.

#### 2.3.3. Measurement of Indicators under Rumen Fermentation Status

Upon opening the bottle, the fermentation fluid was filtered through a nylon bag into a beaker, and the pH was immediately measured using a pH meter (Testo, Schwarzwald, Germany). Subsequently, the fermentation fluid was aliquoted into five 2 mL cryogenic vials and stored at −80 °C for VFA and microbial analysis. Ammonia nitrogen (NH_3_-N) was measured following the method described by Weatherburn [20].

#### 2.3.4. Determination of VFAs in Rumen Fluid

VFAs in rumen fluid samples were analyzed using an ion chromatograph from Sykam, (Sykam, Munich, Germany). The procedure was as follows: First, rumen fluid samples were thawed at 4 °C and centrifuged at 4000 rpm for 10 min. In a 1.5 mL centrifuge tube, 1 mL of the supernatant was accurately mixed with 0.2 mL of metaphosphoric acid solution (25% concentration), vortexed thoroughly, and allowed to stand at 4 °C for over 30 min. After another centrifugation at 10,000 rpm for 10 min, 10 µL of the clear supernatant was diluted 100-fold in a new 1.5 mL centrifuge tube, filtered through a 0.22 µm pore size filter, and then analyzed. The ion chromatograph was operated under the following conditions: Dionex AS11-HC column (4 × 250 mm; Thermo Fisher Scientific, Waltham, MA, USA) at a column temperature of 30 °C and a flow rate of 1.6 mL/min, with an injection volume of 50 µL. A dual-concentration eluent system was used for chromatographic separation: a 0.5 mmol/L NaOH solution was maintained for 25 min, followed by a 50 mmol/L NaOH solution for 5 min, and finally, a 0.5 mmol/L NaOH solution for 10 min. The concentrations of acetate, propionate, and butyrate in the samples were quantified using a standard curve method.

#### 2.3.5. Determination of Methanogen Diversity in Rumen

Total microbial DNA was extracted using the E.Z.N.A.^®^ soil DNA kit (Omega Bio-tek, Norcross, GA, USA). Methanogen diversity was assessed by PCR amplification using primers MLfF (5′-GGTGGTGTMGGATTCACACARTAYGCWACAGC-3′) and MLfR (5′-TTCATTGCRTAGTTWGGRTAGTT-3′) [21]. The PCR conditions were as follows: initial denaturation at 95 °C for 3 min, followed by 27 cycles of 95 °C for 30 s, 55 °C for 30 s, and 72 °C for 30 s, with a final extension at 72 °C for 10 min and storage at 4 °C. The PCR reaction mixture included 4 μL of 5× TransStart FastPfu buffer, 2 μL of 2.5 mM dNTPs, 0.8 μL of each primer (5 μM), 0.4 μL of TransStart FastPfu DNA polymerase, 10 ng of template DNA, and water to a final volume of 20 μL. Each sample was replicated three times.

#### 2.3.6. Illumina MiSeq Sequencing

Library preparation was performed using the NEXTFLEX^®^ Rapid DNA-Seq Kit. Libraries that passed quality control were sequenced on the Illumina MiSeq PE300 platform.

#### 2.3.7. Methods for Determining the Diversity of Rumen Protozoa

The procedure for assessing rumen protozoa diversity followed the same steps as in Section 2.3.6. The primers used for protozoa diversity were RP841F (5′-GACTAGGGATTGGARTGG-3′) and Reg1320R (5′-AATTGCAAAGATCTATCCC-3′) [7].

#### 2.3.8. Data Processing 

Raw sequencing sequences were quality-controlled using Trimmomatic software (http://www.usadellab.org/cms/index.php?page=trimmomatic (accessed on 10 December 2020)). Assembly of paired-end reads based on their overlaps was conducted using FLASH software (V1.2.7, http://ccb.jhu.edu/software/FLASH/ (accessed on 10 December 2020)) [22]. OTU clustering and chimera removal were performed using the UPARSE software (version 7.1, http://drive5.com/uparse/ (accessed on 10 December 2020)) [23], with a similarity threshold of 97%. Species annotation for each sequence was carried out using the RDP classifier (http://rdp.cme.msu.edu/ (accessed on 12 December 2020)), comparing against the nt database with a matching threshold set at 70%.

### 2.4. Animal Experiment Design

This study involved Holstein cows equipped with permanent ruminal cannulas. This experiment was divided into two groups: the control group and the tea polyphenol group, with three cannulated cows in each group. The control group was fed a basal diet, while the tea polyphenol group received a diet supplemented with 1% DM of tea polyphenols (dosage determined from in vitro culture experiments). Adaptation period of 14 days. The trial lasted for 28 days, with blood, ruminal fluid, and ruminal epithelial samples collected on day 29. Throughout the trial, daily feed intake was observed and recorded, with cows fed equal amounts of diet twice daily at 06:00 and 18:00, and water was available ad libitum.

### 2.5. Measurement Indicators and Methods for Animal Experiments 

#### 2.5.1. Determination of Bovine Blood Biochemical Indicators and Antioxidant Indicators 

Blood samples were collected from the caudal vein of each cow before the morning feeding on day 29 of the trial. About 10 mL of blood was drawn from each cow and centrifuged at 3000 rpm for 10 min to obtain serum. Serum biochemical parameters, including alanine aminotransferase (ALT), aspartate aminotransferase (AST), glucose (GLU), triglycerides (TG), cholesterol (CHO), low-density lipoprotein cholesterol (LDL), high-density lipoprotein cholesterol (HDL), and blood urea nitrogen (BUN) were measured using a Chemray 240 automatic biochemistry analyzer (Rayto Life and Analytical Sciences, Shenzhen, China).

The antioxidant capacity of the blood, including total antioxidant capacity (T-AOC), glutathione peroxidase (GSH-Px), superoxide dismutase (SOD), and malondialdehyde (MDA), was assessed using assay kits from Nanjing Jiancheng Bioengineering Institute.

#### 2.5.2. Proteomic Analysis for Bovine Rumen Epithelium

Two hours after the morning feeding on day 29, ruminal papillae were collected from each cow through the ruminal cannula, with 20 papillae collected per cow. Samples were immediately frozen in liquid nitrogen and stored at −80 °C in the laboratory for subsequent analysis.

Total protein was extracted using urea lysis buffer (8 M urea, 1% SDS) containing protease inhibitors that can inhibit various types of proteases and aminopeptidases. Samples were homogenized three times for 40 s each in a high-throughput tissue grinder, followed by lysis on ice for 30 min with intermittent vortexing every 5 min. After centrifugation at 12,000× *g* at 4 °C for 30 min, the supernatant was collected. Protein concentrations were determined using the bicinchoninic acid (BCA) Protein Assay Kit (Thermo Fisher Scientific, Waltham, MA, USA). For the assay, 4 μL of the sample was mixed with 16 μL of distilled water and 200 μL of BCA solution and added to a 96-well plate. The mixture was incubated at 37 °C for 30 min, and the absorbance was read at 562 nm.

Initially, 100 µg of protein was mixed with TEAB to achieve a final concentration of 100 mM. TCEP was added to a final concentration of 10 mM and incubated at 37 °C for 60 min. Subsequently, IAM was added to a final concentration of 40 mM, and the mixture was incubated in the dark at room temperature for 40 min. The proteins were precipitated by adding precooled acetone (ratio of acetone to sample volume = 6:1) and incubated at −20 °C for 4 h. After centrifugation at 10,000× *g* for 20 min, the pellet was dissolved in 100 µL of 100 mM TEAB and digested with trypsin using a 1:50 enzyme-to-protein mass ratio overnight at 37 °C.

The Tandem Mass Tag (TMT) reagent (No. A44522, Thermo Fisher, USA) was thawed at room temperature. Each sample was labeled with one tube of TMT reagent per 100 µg of peptide (labels included CON1-113, CON1-114, CON1-117, TP1-118N, TP2-119, and TP3-121) and incubated at room temperature for 2 h. Hydroxylamine was added, and the reaction continued at room temperature for 30 min. The labeled products were combined equally and concentrated using a vacuum concentrator. Finally, all samples were pooled and vacuum-dried.

For enhanced proteomic depth, samples were fractionated using high pH reverse-phase liquid chromatography. Peptide samples were resolubilized in UPLC loading buffer (2% acetonitrile, adjusted to pH 10 with ammonia) and separated using an ACQUITY UPLC BEH C18 column (1.7 µm, 2.1 mm × 150 mm, Waters, Milford, MA, USA). The separation was achieved with a gradient of elution consisting of 2% acetonitrile (Phase A) and 80% acetonitrile (Phase B), both at pH 10, over 48 min at a flow rate of 200 μL/min. The peptides were eluted using the following gradient: 0~1.9 min, 0~0% B; 1.9~2 min, 0~5% B; 2~17 min, 5~5% B; 17~18 min, 5~10% B; 18~35.5 min, 10~30% B; 35.5~38 min, 30~36% B; 38~39 min, 36~42% B; 39~40 min, 42~100%B; 40~44 min, 100% B; 44~45 min, 100~0% B; 45~48 min, 0% B. A detailed gradient schedule was followed to optimize peptide elution. In total, 28 fractions were collected, optimized by peak shape and time, combined into 14 fractions, and concentrated by vacuum centrifugation for further analysis.

The peptides were analyzed using a two-dimensional liquid chromatography–tandem mass spectrometry setup, combining Evosep One with an Orbitrap Exploris 480 mass spectrometer (Majorbio Bio-Pharm Technology Co., Ltd., Shanghai, China). 

Separation was conducted on a C18 column (150 μm × 15 cm, Evosep, Denmark) using a linear gradient of solvent A (water with 0.1% formic acid) and solvent B (100% acetonitrile (ACN) with 0.1% formic acid) at a flow rate of 300 nL/min. The peptides were eluted using the following gradient: 0~2 min, 5~5% B; 2~30 min, 5~38% B; 30~40 min, 38~90% B; 40~44 min, 90~90% B.

The Orbitrap Exploris 480 operated in data-dependent acquisition mode (DDA), alternating between full scan MS and MS/MS acquisition. Full-scan MS spectra were captured in the range of *m*/*z* 350–1500 at a resolution of 60K. Selected precursor ions were fragmented using higher-energy collision dissociation (HCD) with MS/MS resolution set at 15K. Dynamic exclusion was applied for 30 s to enhance detection efficiency.

Proteomic data were processed using ProteomeDiscoverer (Version 2.4, Thermo Scientific, Waltham, MA, USA) against the Uniprot database. Settings included a Precursor Mass Tolerance of 20 ppm and Fragment Mass Tolerance of 0.02 Da. The identification process was stringent, with a false discovery rate (FDR) threshold of ≤0.01 and a requirement for at least one unique peptide per protein identification.

Protein annotation involved the use of Gene Ontology (GO) and Kyoto Encyclopedia of Genes and Genomes (KEGG) databases, and DEPs were subjected to further GO and KEGG enrichment analysis to elucidate biological functions and pathways involved.

#### 2.5.3. Verification of Differential Proteins by PRM Protein Relative Quantification 

To validate the findings from TMT proteomics, 10 target proteins were selected for analysis using liquid chromatography–parallel reaction monitoring (LC-PRM) mass spectrometry. Initially, proteins from the samples were extracted, enzymatically digested into peptides, and desalted prior to quantification. Equal amounts of these digested peptides were then prepared in mass spectrometry loading buffer (2% acetonitrile with 0.1% formic acid) and analyzed using an EASY-nLC 1200 system coupled with a Q Exactive HF-X quadrupole orbitrap mass spectrometer (Thermo Fisher Scientific, Waltham, MA, USA) at Majorbio Bio-Pharm Technology Co., Ltd. (Shanghai, China). Peptides were separated on a C18-reversed phase column (75 µm × 25 cm, Thermo Fisher Scientific, Waltham, MA, USA) using an equilibrated solvent system of 2% ACN with 0.1% formic acid (solvent A) and 80% ACN with 0.1% formic acid (solvent B). The elution was performed with a gradient increasing from 5% to 100% solvent B over 120 min at a flow rate of 300 nL/min. The peptides were eluted using the following gradient: 0–64 min, 5–23% B; 64–80 min, 23–29%B; 80–90 min, 29–38% B; 90–92 min, 38–48% B; 92–93 min, 48–100% B; 93–120 min, maintain 100%B. The eluted peptides were detected using a Q-Exactive HF-X mass spectrometer in positive ion mode, employing PRM with HCD for fragmentation. The full scan range was set from *m*/*z* 350 to 1500 with a resolution of 60,000. PRM scans were subsequently acquired with a secondary resolution of 15,000 and a normalized collision energy of 28%. Dynamic exclusion was set to 18 s to prevent redundant sampling of abundant ions.

For PRM data analysis, Skyline software (3.5.0 version) was utilized to extract and manually validate peak data from the raw PRM output. For quantitative analysis, 3–4 of the most abundant ions from each peptide were selected, focusing on fragments from y3 to y(n − 1). The peak area data for each target peptide were extracted and compiled, and the sub-ion peak areas were integrated for comprehensive analysis. Furthermore, the quantitative peptide values from the target proteins were normalized based on labeled peptides and subjected to statistical analysis using Student’s *t*-test, with a significance threshold set at *p* < 0.05. Comparisons were made between the quantitative values obtained from label-free or TMT proteomics and the PRM validation results to confirm consistency and accuracy in protein quantification.

### 2.6. Statistical Analysis

Proteins statistical analysis of the data was performed on the Majorbio I-Sanger Cloud platform (cloud.majorbio.com), using thresholds of fold change (>1.2 or <0.83) and *p*-value < 0.05 for identifying differentially expressed proteins (DEPs).

The analyses were performed using the software (9.4 version, SAS Institute Inc., Cary, NC, USA). The fermentation parameters data were repeatedly measured using a mixed model with tea polyphenols treatment as fixed effects and run and the run×tea polyphenols treatment interaction as random effects. *t*-test was used to analyze other data. Results are presented as mean and standard error, with *p* < 0.05 considered statistically significant, and the level of significance considered for the tendencies was 0.05 < *p* < 0.10.

## 3. Results

### 3.1. In Vitro Experiment Results

#### 3.1.1. Effects of Tea Polyphenols on CH_4_ Production in Bovine Rumen In Vitro

As shown in Table 1, compared to the control group, tea polyphenols significantly reduced both GP and CH_4_ production at all addition levels (*p* < 0.05). At a 4% addition level, tea polyphenols significantly decreased the NH_3_-N content (*p* < 0.05). Based on a comprehensive analysis of the effect of tea polyphenols on methane generation, the optimal addition level of 1% tea polyphenols was chosen for subsequent experiments.

#### 3.1.2. Impact of Tea Polyphenols on VFA Production in Bovine Rumen In Vitro

According to Table 2, tea polyphenols did not significantly affect the concentrations of acetate, propionate, and butyrate (*p* > 0.05), but they significantly lowered the acetate/propionate ratio (*p* < 0.05).

#### 3.1.3. Effects of Tea Polyphenols on Methanogen Diversity in Bovine Rumen In Vitro

Methanogenic diversity was analyzed using the PE300 platform, yielding a total of 119,431,984 bases from 10 rumen fluid samples, which, after optimization, resulted in 82,274,515 bases with an average sequence length of 414 bp. As depicted in Supplementary S4: Figure S2, the tea polyphenols affected alpha diversity indices such as Chao, Ace, Shannon, and Simpson but without significant differences (*p* > 0.05). Supplementary S3: Figure S1 illustrates a clear separation between the control and tea polyphenol groups, indicating that the tea polyphenol treatment altered the community structure of the rumen methanogens.

#### 3.1.4. Analysis of the Impact of Tea Polyphenols on the Community Structure and Species Abundance of Methanogens in Bovine Rumen In Vitro 

**Figure 1:**
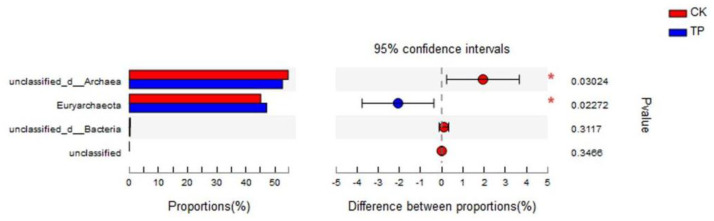
Analysis of rumen methanogens community structure at the phylum level in in vitro fermentation. CK: control group; TP: tea polyphenols.

At the phylum taxonomic level (Supplementary S5: Figure S3), this study identified four methanogen phyla, predominantly composed of *unclassified_d_Archaea* and *Euryarchaeota*, together accounting for over 99% of the relative abundance. Species differential analysis (Figure 1) revealed that tea polyphenols significantly reduced the relative abundance of *unclassified_d_Archaea* (*p* < 0.05) and significantly increased the relative abundance of *Euryarchaeota* (*p* < 0.05).

#### 3.1.5. Genus-Level Analysis of Methanogen Composition

**Figure 2:**
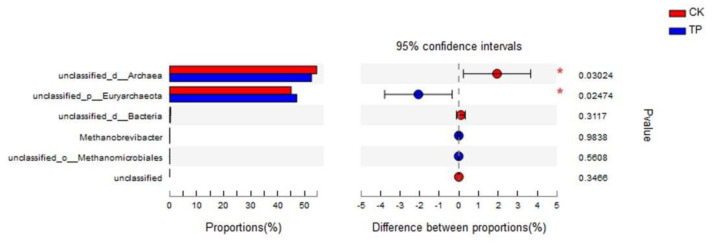
Analysis of rumen methanogens community structure at the genus level in in vitro fermentation. CK: control group; TP: tea polyphenols.

At the genus taxonomic level (Supplementary S6: Figure S4), six methanogen genera were identified, primarily consisting of *unclassified_d_Archaea* and *unclassified_p Euryarchaeota*, with their combined relative abundances also exceeding 99%. Species differential analysis (Figure 2) showed that tea polyphenols significantly reduced the relative abundance of *unclassified_d_Archaea* in the rumen (*p* < 0.05) and significantly increased the relative abundance of *unclassified_p Euryarchaeota* (*p* < 0.05).

**Figure 3:**
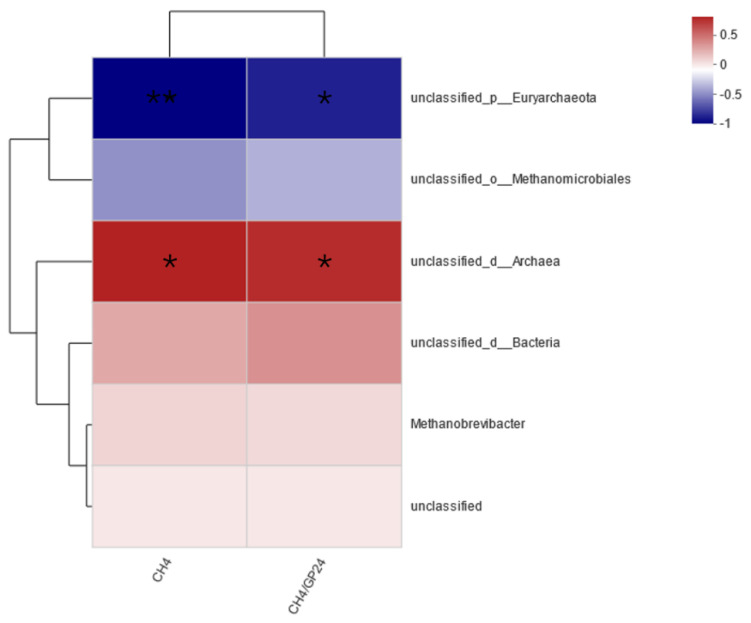
Spearman’s correlation analysis between the relative abundance of dominant methanogens genus and environmental factor in in vitro fermentation. * 0.01 < *p* ≤ 0.05, ** 0.001 < *p* ≤ 0.01.

Spearman’s correlation analysis relating CH_4_ concentration and production to the relative abundances of different methanogen genera demonstrated (Figure 3) that *unclassified_p Euryarchaeota* was negatively correlated with both CH_4_ production and concentration (*p* < 0.05), whereas *unclassified_d_Archaea* showed a positive correlation with both (*p* < 0.05).

#### 3.1.6. Effects of Tea Polyphenols on Protozoan Diversity in Bovine Rumen Fermentation In Vitro

In this study, protozoa were sequenced using the PE300 platform, yielding a total of 102,533,242 bases from 10 rumen fluid samples, which, after optimization, resulted in 80,680,373 bases with an average sequence length of 473 bp. As depicted in Supplementary S8: Figure S6, the tea polyphenols did not significantly affect the alpha diversity indices such as Chao, Ace, Shannon, and Simpson (*p* > 0.05). However, Supplementary S7: Figure S5 illustrates a distinct separation between the control and tea polyphenol groups, suggesting that the tea polyphenols exerted an influence on the community structure of the rumen protozoa.

#### 3.1.7. Analysis of the Impact of Tea Polyphenols on Protozoan Community Structure and Species Abundance 

At the phylum taxonomic level (Supplementary S9: Figure S7), this study identified four protozoan phyla, predominantly composed of *unclassified_d_Eukaryota* and *Ciliophora*, which together accounted for over 99% of the relative abundance. At the genus level (Supplementary S10: Figure S8), ten protozoan genera were identified, mainly consisting of *unclassified_d_Eukaryota*, *Eodinium*, *Pseudoentodinium*, *Epidinium*, *Entodinium*, *Balantioides*, and *Ostracodinium*. Overall, the addition of tea polyphenols influenced the composition of protozoa at both the phylum and genus levels.

**Figure 4:**
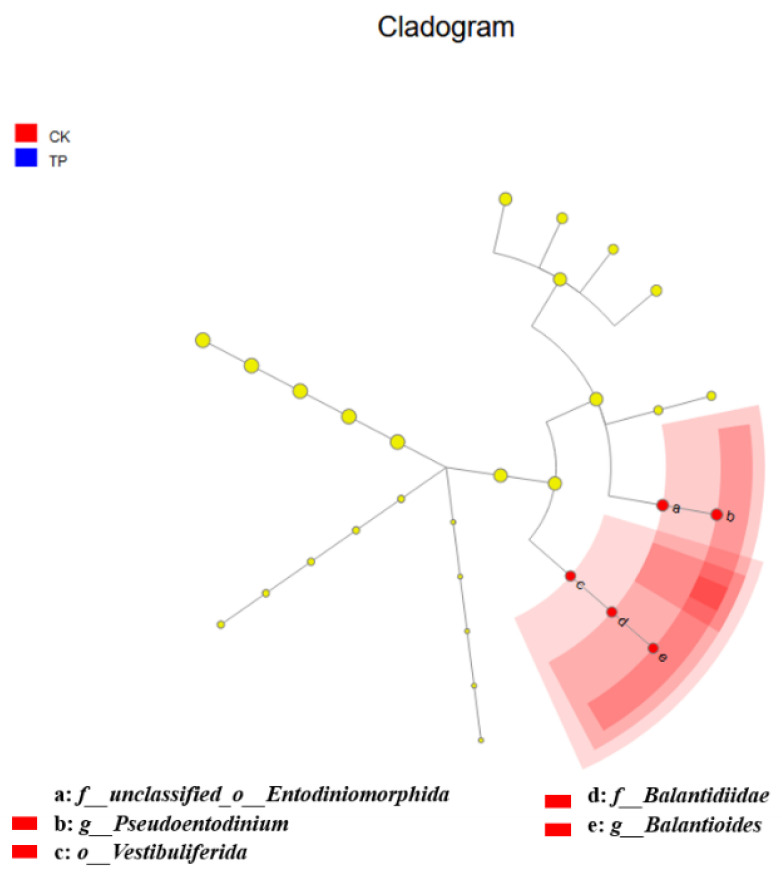
Analysis of rumen protozoa community structure from phylum to genus level in in vitro fermentation. CK: control group; TP: tea polyphenols.

LEfSe analysis identified significant differences in protozoan populations between the control and tea polyphenol groups, as shown in Figure 4. Compared to the tea polyphenol group, the control group’s rumen fluid samples were significantly enriched with protozoa such as *f__unclassified_o__Entodiniomorphida*, *f__Balantidiidae*, *o__Vestibuliferida*, *g__Pseudoentodinium*, and *g__Balantioides*.

#### 3.1.8. Correlation between CH_4_ Production and Concentration with Protozoa

**Figure 5:**
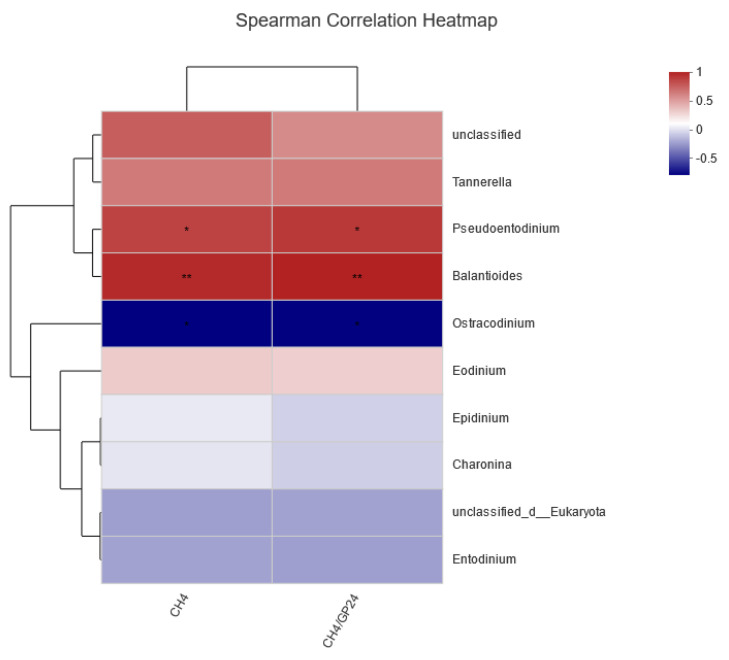
Spearman’s correlation analysis between the relative abundance of dominant protozoa genus and environmental factor in in vitro fermentation. * 0.01 < *p* ≤ 0.05, ** 0.001 < *p* ≤ 0.01.

Spearman’s correlation analysis was conducted to assess the relationship between the CH_4_ concentration, production, and the relative abundances of different protozoan genera (Figure 5). The results indicated that *Pseudoentodinium* was positively correlated with both CH4 production and concentration (*p* < 0.05). Similarly, *Balantioides* showed a significant positive correlation with both CH_4_ production and concentration (*p* < 0.05). In contrast, *Ostracodinium* was significantly negatively correlated with both CH_4_ production and concentration (*p* < 0.05).

### 3.2. Animal Experiment Results

#### 3.2.1. Effects of Tea Polyphenols on Bovine Blood Biochemical and Antioxidant Indicators

As shown in Table 3, the addition of tea polyphenols to the diet did not significantly affect biochemical indicators such as ALT, AST, and BUN in bovine blood (*p* > 0.05). Table 4 indicates that dietary supplementation with tea polyphenols significantly increased the levels of T-AOC and GSH-PX in bovine blood (*p* < 0.05) but had no significant effect on the levels of SOD and MDA (*p* > 0.05).

#### 3.2.2. Effects of Tea Polyphenols on Rumen Fermentation Parameters in Dairy Cows

According to Table 5, the addition of tea polyphenols to the diet significantly reduced the concentration of NH_3_-N in rumen fluid (*p* < 0.05). There was a trend towards increased propionate concentrations and reduced acetate/propionate ratios in the rumen fluid with tea polyphenol supplementation (0.05 < *p* < 0.1), although there were no significant effects on the concentrations of acetate and butyrate (*p* > 0.05).

#### 3.2.3. Impact of Tea Polyphenols on the Rumen Microbiota of Dairy Cows

**Figure 6:**
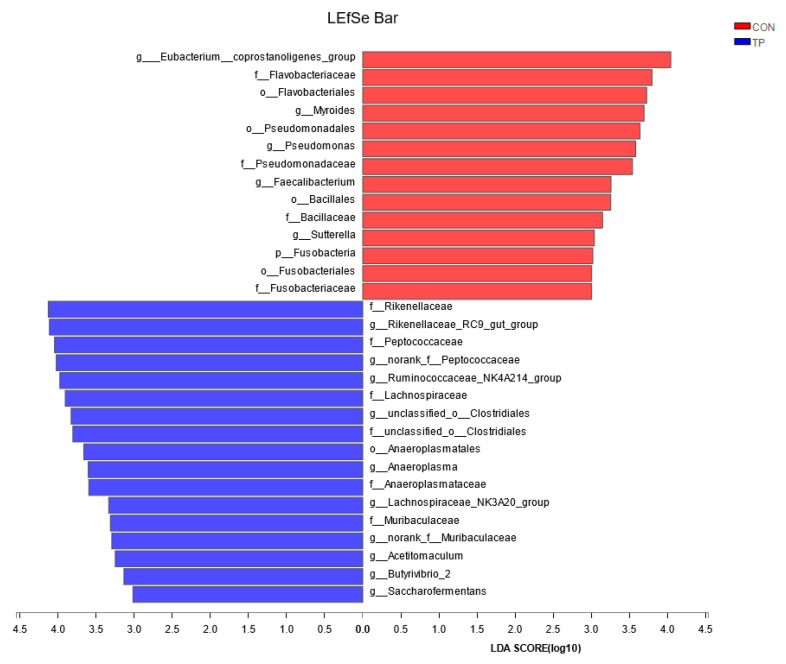
Linear discriminant analysis effect size results of the main microbes. CON: control group; TP: tea polyphenols.

The rumen microbiota was sequenced using the PE300 platform, resulting in a total of 279,739,768 bases from six rumen fluid samples. After optimization and selection, 192,175,674 bases were retained with an average sequence length of 414.56 bp. LEfSe differential analysis (Figure 6) revealed significant enrichment of microbes such as *Rikenellaceae_RC9_gut_group*, *Ruminococcaceae_NK4A214_group*, and *Butyrivibrio_2* in the tea polyphenol group compared to the control group, which showed significant enrichment of *Fusobacterium*.

#### 3.2.4. Correlation between Rumen Fermentation Parameters and Microbiota

**Figure 7:**
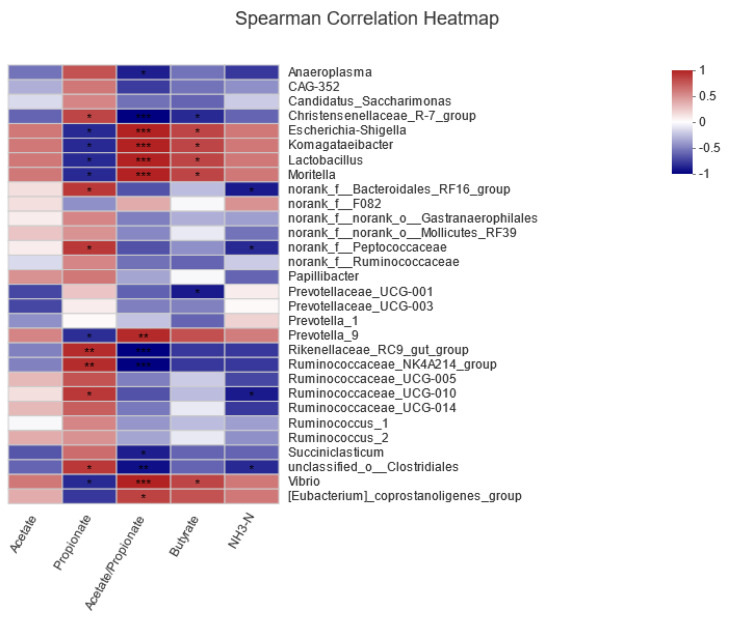
Spearman’s correlation analysis between the relative abundance of the dominant genus (TOP 30) and environmental factors. * 0.01 < *p* ≤ 0.05, ** 0.001 < *p* ≤ 0.01, *** *p* ≤ 0.001.

Spearman’s correlation analysis was performed to assess the relationships between rumen fermentation parameters and microbiota (Figure 7). The analysis indicated that *Anaeroplasma* was significantly negatively correlated with the acetate/propionate ratio (*p* < 0.05). *Escherichia-Shigella*, *Komagataeibacter*, *Lactobacillus*, *Vibrio*, and *Moritella* were significantly negatively correlated with propionate (*p* < 0.05) but positively correlated with the acetate/propionate ratio and butyrate (*p* < 0.05). Both *Rikenellaceae_RC9_gut_group* and *Christensenellaceae_R-7_group* showed significant positive correlations with propionate and negative correlations with the acetate/propionate ratio (*p* < 0.05).

#### 3.2.5. Proteomics Analysis of Rumen Epithelium in Dairy Cows 

##### Protein Information Statistics

In this study, a total of 442,507 spectra were identified, with 99,230 spectra matched. These matched spectra identified 46,393 peptides, corresponding to 6051 proteins. 

##### Selection of Differentially Expressed Proteins in Rumen Epithelium

**Figure 8:**
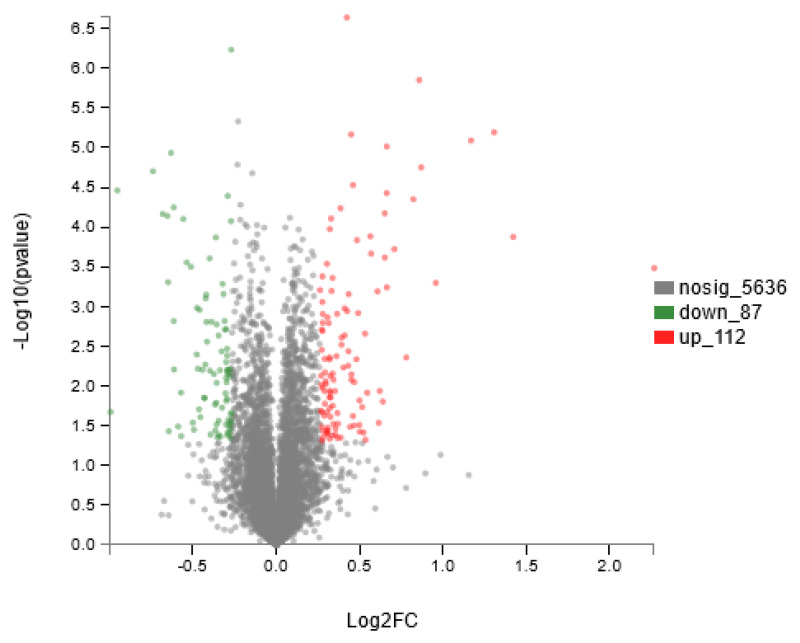
Volcano plot analysis of differentially expressed proteins in rumen epithelium.

Differential proteins between the control and tea polyphenol groups were selected using iTRAQ, as shown in Figure 8. Proteins were filtered through fold change (FC) and *t*-test criteria; proteins were considered upregulated if FC > 1.2 and *p* < 0.05 and downregulated if FC < 0.83 and *p* < 0.05. A total of 199 differential proteins were identified (Figure 8), comprising 112 upregulated and 87 downregulated proteins.

##### GO Functional Analysis of Differentially Expressed Proteins in Rumen Epithelium

**Figure 9:**
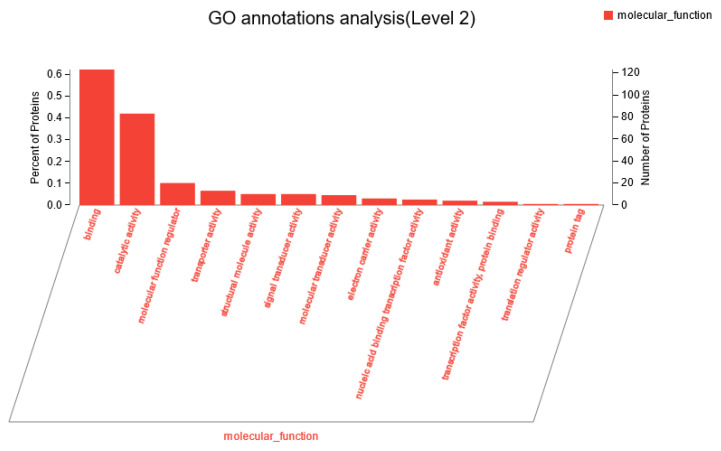
Molecular function of differentially expressed proteins in rumen epithelium cells.

Supplementary S11: Figure S9, Supplementary S12: Figure S10, and Figure 9 display the top 20 GO terms associated with differentially expressed proteins in the rumen epithelium of dairy cows fed diets supplemented with tea polyphenols, categorized into biological processes, cellular components, and molecular functions. Results from Supplementary S11: Figure S9 indicated that the addition of tea polyphenols involved differential proteins in cellular processes, single-organism processes, metabolic processes, biological regulation, response to stimuli, immune system processes, and growth compared to the control group. As depicted in Supplementary S12: Figure S10, differentially expressed proteins were primarily located in cellular components such as the cell parts, organelles, membranes, and macromolecular complexes. Additionally, these proteins exhibited molecular functions, including binding, catalytic activity, transporter activity, and antioxidant activity (Figure 9).

##### PRM Validation of Differential Proteins 

**Figure 10:**
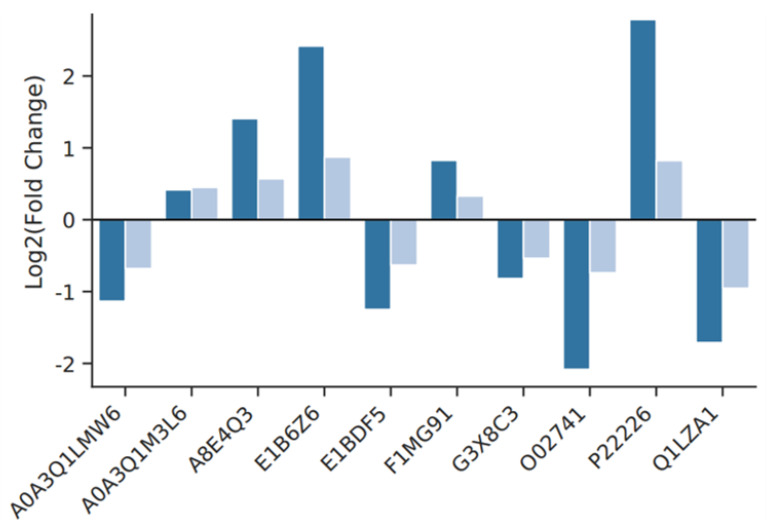
Comparison of results between PRM and Proteome in rumen epithelium cells. The dark blue bar chart represents the results of PRM, the light blue bar chart represents the results of proteomics.

Supplementary S13: Figure S11 presents the mass spectra obtained during PRM validation. The validation results revealed that the expression trends of the 10 proteins analyzed were consistent with the proteomics findings (Figure 10), confirming the reliability and accuracy of the proteomics results.

##### Correlation Analysis between Rumen Microbial Metabolites and Rumen Epithelium Transport Proteins

**Figure 11:**
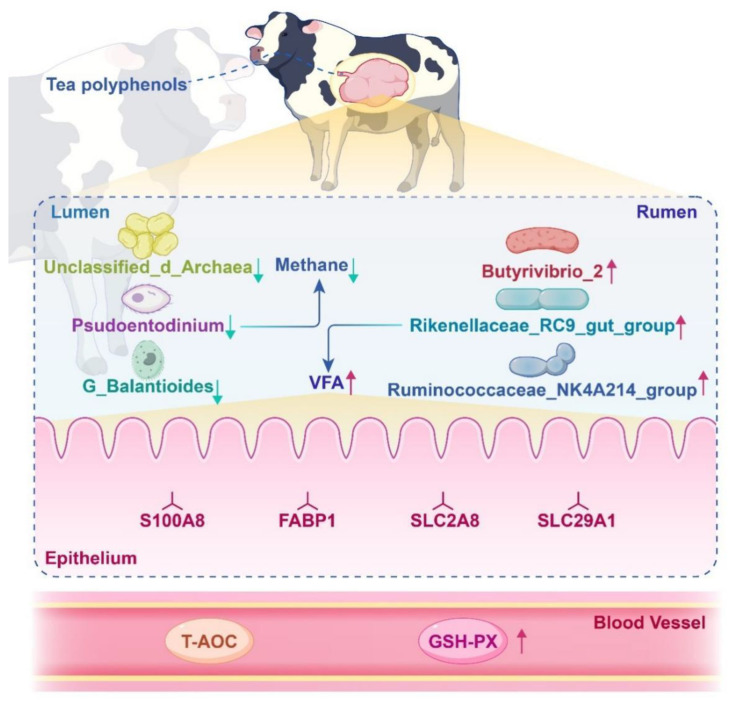
Proposed model of tea polyphenols inhibits methanogenesis and improves rumen epithelial transport in dairy cows. The red arrow represents promotion, and the green arrow represents inhibition.

Further analysis focused on the proteins involved in transporter activity identified in the GO analysis. Changes in these differentially expressed proteins are shown in Table 6. Using Spearman’s correlation analysis, relationships were examined between these transporter proteins and rumen fermentation parameters, as detailed in Table 7. The results indicated that dietary supplementation with tea polyphenols promoted the expression of the FABP1 protein (*p* < 0.05), which was significantly positively correlated with propionate levels (*p* < 0.05). Conversely, the expression of proteins RAB4A and LOC107131172 was reduced (*p* < 0.05); RAB4A was negatively correlated with propionate levels (*p* < 0.05) and positively correlated with NH3-N levels (*p* < 0.05), while LOC107131172 showed a positive correlation with the acetate/propionate ratio (*p* < 0.05). Based on the results of this article, we have drawn proposed model diagram of tea polyphenols inhibits methanogenesis and improves rumen epithelial transport in dairy cows (Figure 11).

## 4. Discussion

This study, through in vitro and in vivo experiments and employing multi-omics approaches, explores the effects of tea polyphenols on CH_4_ production and VFA transport in bovine rumen epithelial cells, aiming to clarify the intrinsic relationships between tea polyphenols, the rumen microbiome, and the host. Our in vitro studies indicated that tea polyphenols could decrease total GP and CH_4_ output, significantly reducing the acetate/propionate ratio. The CH_4_ production decreased from 2.27 mL in 0% to 1.68 mL in 1%, and the acetate/propionate ratio decreased from 3.95 in 0% to 3.52 in 1%. During rumen fermentation, acetate production is accompanied by hydrogen generation, and high hydrogen partial pressures may inhibit the degradation of feed [24], whereas propionate production consumes hydrogen, indicating that propionate formation in the rumen can reduce the substrates available for CH_4_ production [25,26]. Studies have shown that monensin can reduce CH_4_ emissions by lowering the rumen acetate/propionate ratio [27,28]. CH_4_ production correlates positively with the rumen acetate/propionate ratio, and the reduction in this ratio partly explains the decreased CH_4_ production due to tea polyphenols.

Rumen methanogens play a critical role in CH_4_ formation, converting CO_2_, H_2_, methanol, and formate into CH_4_ [29]. Methanogens produce CH_4_ primarily through the catalysis of the mcr enzyme complex [30], accounting for over 90% of total CH_4_ generation. Our findings revealed that tea polyphenols significantly reduced the relative abundance of *unclassified_d_Archaea* at the phylum and genus levels. Spearman’s correlation analysis showed a significant positive correlation between *unclassified_d_Archaea* and both CH_4_ production and concentration, suggesting that tea polyphenols can affect the abundance of methanogens, thereby influencing CH_4_ generation. Jayanegara et al. (2015) found that tannins in vitro could reduce the number of methanogens [31] due to tannins binding with proteins or microbial cell enzymes to inhibit methanogen activity [32,33]. These studies confirm that polyphenolic compounds (both tannins and non-tannins) can affect the quantity and community structure of methanogens, thereby impacting CH_4_ production.

A symbiotic relationship exists between rumen protozoa and methanogens, where methanogens can attach to the surface or inside of protozoa [34,35]. Consequently, the composition of rumen protozoa affects the production of CH_4_ in ruminants. The LEfSe differential analysis in this study showed that tea polyphenols influenced the community structure of rumen protozoa, significantly enriching protozoa such as *f__unclassified_o__Entodiniomorphida*, *f__Balantidiidae*, *o__Vestibuliferida*, *g__Pseudoentodinium*, and *g__Balantioides* in the control group. Spearman’s correlation analysis indicated a positive correlation between *Balantioides* and both CH_4_ production and concentration, suggesting that tea polyphenols reduce CH_4_ production by inhibiting the activity of *Balantioides* protozoa in the rumen. Poungchompu et al. (2009) examined the impact of concentrated tannins from mangosteen pericarp and soapnut fruit on the number of rumen protozoa, showing that these tannins significantly reduced the numbers of both methanogens and protozoa in rumen fluid [36]. In summary, the findings suggest that tea polyphenols reduce CH_4_ production by decreasing the numbers of both methanogens and protozoa, though a large proportion of methanogens and protozoa highly correlated with CH_4_ production remain unidentified in this study. Further functional observations of these microbes are needed to elucidate the microbial mechanisms by which tea polyphenols affect CH_4_ production in the rumen.

The in vivo experiments suggested a trend toward increased rumen liquid propionate concentration and a reduced acetate/propionate ratio. This is consistent with the results of the in vitro experiments. Further investigation using 16S high-throughput sequencing revealed significant enrichment of the *Ruminococcaceae_NK4A214_group* in the tea polyphenol group. Typically, a higher proportion of concentrate in the diet favors propionate fermentation. Research comparing the effects of high-concentrate and high-forage diets on rumen microbiota found significant enrichment of the *Ruminococcaceae_NK4A214_group* in high-concentrate diets, significantly associated with VFA production [37]. Thus, tea polyphenols may alter the composition of VFAs in the rumen of dairy cows by modulating the relative abundance of rumen microbiota. The LEfSe differential analysis in this study revealed significant enrichment of microbes such as the *Rikenellaceae_RC9_gut_group*, *Anaeroplasma*, *Lachnospiraceae_AC2044_group*, and *Butyrivibrio_2* in the tea polyphenol group compared to the control. Significant enrichment of the *Lachnospiraceae_AC2044_group* in the tea polyphenol group suggested that consumption of tea polyphenols may promote fiber degradation in dairy cows, but the specific impact mechanism still needs further exploration. Furthermore, studies correlating microbes with feed efficiency have shown significant associations between *Anaeroplasma* and *Butyrivibrio_2* with feed efficiency [38], further suggesting that adding tea polyphenols to the diet may enhance the feed efficiency of the animals.

Rumen microbial fermentation of feed results in the production of VFAs, which constitute 70–80% of the absorbed energy for ruminants [39]. The rumen epithelium serves as a unique interface for interactions between host and microbial metabolisms, with the absorption capabilities of VFAs in the rumen epithelium influencing the net utilization of nutrients in the organism. This study conducted a GO analysis on the differential proteins in the rumen epithelial cells of dairy cows fed with tea polyphenols, finding these proteins enriched in transporter activity molecular functions. Compared to the control group, proteins such as COIII, S100A8, FABP1, SLC2A8, and SLC29A1 were significantly upregulated, whereas HBB, RAB4A, RBP4, LOC107131172, HBA, and ZFYVE19 were downregulated in the tea polyphenol group. S100A8 can specifically form a complex with polyunsaturated fatty acid arachidonic acid (AA), known as S100A8-AA, which then acts as a transport protein to deliver arachidonic acid to target cells [40]. The solute carrier family (SLC) represents one of the most important families of membrane transport proteins, involved in intercellular material transport, nutrient metabolism, and energy transfer [41]. In this study, significant upregulation of SLC2A8 and SLC29A1 was observed in the rumen epithelium, indicating that dietary inclusion of tea polyphenols enhances the expression of nutrient transport proteins in rumen epithelial cells.

Fatty acid-binding proteins (FABPs), including FABP1, FABP2, and FABP6, function in the intestine. FABPs can bind to fatty acids and other hydrophobic ligands, transporting them to locations for fatty acid oxidation or triglyceride synthesis [42]. Additionally, FABPs regulate biochemical processes, particularly lipid metabolism, by modulating fatty acid concentrations [43]. Studies have shown that in mammals, FABP1 can transport fatty acids via aqueous diffusion [44]. In this experiment, compared to the control group, FABP1 protein was significantly upregulated in the tea polyphenol group and positively correlated with the concentration of propionate in the rumen. This suggests that dietary tea polyphenols enhance the transport capacity of VFAs in rumen epithelial cells.

The absorption and transport of nutrients in animals require the mediation of blood, which contains various biochemical markers that are closely related to the physiological status of the body. Thus, changes in blood biochemical markers can indicate the transport and utilization of nutrients. Studies have found that phenolic compounds produced by the degradation of tannic acid are transported via the bloodstream to the liver, and excessive amounts beyond the liver’s detoxification capacity can cause moderate symptoms in animals [45]. The results of this study indicated that the addition of tea polyphenols to the diet did not significantly affect the levels of ALT and AST in the blood of dairy cows, suggesting that adding 1% tea polyphenols to the diet has no adverse effects on the liver. Additionally, this study found that dietary tea polyphenols significantly increased the levels of T-AOC and GSH-PX in dairy cow blood, consistent with previous research during the peripartum period, which showed that dietary tea polyphenols can enhance the levels of T-AOC and GSH-PX in dairy cow blood and improve their antioxidant capacity [46]. This is because tea polyphenols are reductants that can reduce free radicals to more stable compounds, thereby clearing harmful free radicals in the body and enhancing the body’s antioxidant capacity to avoid oxidative damage [12,47]. In summary, supplementing dairy cow diets with tea polyphenols can help improve the utilization efficiency of feed energy and the health status of the cows, contributing to the efficient, green, and healthy development of the dairy industry.

## 5. Conclusions

Tea polyphenols reduce the generation of CH_4_ in the rumen by influencing the diversity and community structure of microbes such as bacteria, protozoa, and methanogens, thereby minimizing the loss of feed energy. By affecting the microbial community in the bovine rumen, tea polyphenols alter the fermentation patterns, thereby enhancing the host’s rumen epithelial cells’ capacity to transport VFAs. In production practice, the use of tea polyphenols can enhance the efficiency of nutrient utilization in dairy cows, thereby improving their productive performance.

## Figures and Tables

**Table 1:** Effects of tea polyphenols on gas production, CH_4_ production, pH, and NH_3_-N, in in vitro fermentation.

Index	Groups					SEM	*p*-Value		
	A (0%)	B (1%)	C (2%)	D (3%)	E (4%)		Treatment	Run	Treatment × Run
GP_24_ (mL)	161.04 ^a^	138.58 ^c^	142.19 ^bc^	143.21 ^bc^	145.91 ^b^	2.94	<0.001	0.276	0.176
CH_4_ (mL)	2.27 ^a^	1.68 ^c^	1.86 ^b^	1.960 ^b^	1.97 ^b^	0.08	<0.001	0.557	0.364
CH_4_/GP_24_ (%)	1.41 ^a^	1.21 ^c^	1.31 ^b^	1.37 ^ab^	1.35 ^ab^	0.04	0.010	0.353	0.791
pH	6.85 ^ab^	6.87 ^a^	6.85 ^ab^	6.82 ^b^	6.83 ^b^	0.02	0.033	0.129	0.457
NH_3_-N (mmol/L)	21.66 ^ab^	23.65 ^a^	24.46 ^a^	23.43 ^a^	17.10 ^b^	2.83	0.120	0.463	0.395

Note: Different letters in the same row mean a significant difference (*p* < 0.05); the same letters in the same row mean no significant difference (*p* > 0.05). GP_24_: total gas production.

**Table 2:** Effects of tea polyphenols on VFA in in vitro fermentation (mmol/L).

Item	CK	TP	SEM	*p*-Value
Acetate	61.77	61.36	1.76	0.819
Propionate	15.72	17.32	0.85	0.096
Acetate/Propionate	3.95	3.52	0.14	0.036
Butyrate	7.69	8.30	0.72	0.426

Note: CK: control group; TP: tea polyphenols group.

**Table 3:** Effects of tea polyphenols on blood biochemical indicators of cows.

Index	CK	TP	SEM	*p*-Value
ALT (U/L)	17.63	16.35	0.75	0.166
AST (U/L)	29.23	30.38	1.23	0.405
BUN (mg/dL)	10.69	10.44	0.41	0.579
GLU (mmol/L)	4.40	4.52	0.15	0.484
TG (mmol/L)	0.35	0.36	0.003	0.954
CHO (mmol/L)	2.55	2.91	0.37	0.101
HDL (mmol/L)	1.16	1.29	0.08	0.180
LDL (mmol/L)	0.32	0.33	0.05	0.849

Note: CK: control group; TP: tea polyphenols group; ALT: alanine aminotransferase; AST: aspartate aminotransferase; BUN: blood urea nitrogen; GLU: glucose; TG: triglycerides; CHO: cholesterol; HDL: high-density lipoprotein cholesterol; LDL: low-density lipoprotein cholesterol.

**Table 4:** Effects of tea polyphenols on blood antioxidative indexes of cows.

Index	CK	TP	SEM	*p*-Value
T-AOC (U/mL)	5.92	7.65	0.45	0.019
GSH-PX (U/mL)	59.20	70.40	3.58	0.035
SOD (U/mL)	244.56	250.63	12.12	0.642
MDA (nmol/mL)	1.85	1.98	0.21	0.568

Note: CK: control group; TP: tea polyphenols group; T-AOC: total antioxidant capacity; GSH-Px: glutathione peroxidase; SOD: superoxide dismutase; MDA: malondialdehyde.

**Table 5:** Effects of tea polyphenols on fermentation parameters in rumen of cows.

Index	CK	TP	SEM	*p*-Value
pH	6.48	6.57	0.75	0.344
NH_3_-N (mmol/L)	16.86	12.90	1.11	0.024
Acetate (mmol/L)	80.62	77.49	3.58	0.461
Propionate (mmol/L)	22.07	24.53	0.97	0.065
Acetate/Propionate	3.66	3.16	0.20	0.064
Butyrate (mmol/L)	13.65	12.35	0.76	0.463

**Table 6:** The fold change of differentially expressed proteins in transporter activity of GO annotations analysis.

Gene Name	Accession No.	Protein Name	Fold Change	Expression
COIII	Q6JTG5	Cytochrome c oxidase subunit 3	1.53	up
HBB	D4QBB4	Globin A1	0.80	down
S100A8	P28782	Protein S100-A8	1.26	up
FABP1	P80425	Fatty acid-binding protein	1.46	up
RAB4A	Q2TBH7	Ras-related protein Rab-4A	0.72	down
SLC2A8	F1MZY9	Solute carrier family 2, facilitated glucose Transporter member 8	1.24	up
RBP4	A0A3Q1MSW9	Retinol-binding protein 4	0.77	down
LOC107131172	G3N1Y3	GLOBIN domain-containing	0.79	down
SLC29A1	F6PZI3	Solute carrier family 29 member 1	1.25	up
HBA	P01966	Hemoglobin subunit alpha	0.81	down
ZFYVE19	Q2HJ64	Zinc finger FYVE-type containing 19	0.80	down

**Table 7:** The correlation between rumen fermentation parameters and the differentially expressed proteins in transporter activity of GO annotations analysis.

Items	Acetate	Propionate	Acetate/Propionate	Butyrate	NH_3_-N
COIII	−0.371	0.771	−0.486	−0.371	−0.771
HBB	0.371	−0.771	0.771	0.771	0.600
S100A8	0.143	0.771	−0.486	−0.371	−0.771
FABP1	−0.200	0.943 *	−0.714	−0.657	−0.771
RAB4A	−0.086	−0.829 *	0.600	0.314	0.886 *
SLC2A8	−0.543	0.600	−0.543	−0.486	−0.600
RBP4	0.486	−0.771	0.657	0.714	0.600
LOC107131172	0.543	−0.771	0.829 *	0.543	0.771
SLC29A1	−0.371	0.771	−0.486	−0.371	−0.771
HBA	0.143	−0.600	0.429	0.429	0.600
ZFYVE19	0.486	−0.771	0.657	0.714	0.600

NOTE: * mean significant correlation (*p* < 0.05).

## Data Availability

The data presented in this study are available upon request from the corresponding author. The data are not publicly available to preserve the privacy of the data.

## Supplementary Materials

The following supporting information can be downloaded at: https://www.mdpi.com/article/10.3390/ani14172569/s1, S1. Table S1. Composition of artificial saliva. S2. Table S2. Ratio of artificial saliva. S3. Figure S1. Effects of tea polyphenols on alpha diversity of methanogens in in vitro fermentation. S4. Figure S2. Effects of tea polyphenols on alpha diversity of methanogens in in vitro fermentation. S5. Figure S3. Effects of tea polyphenols on the composition of methanogens (phylum level) in in vitro fermentation. S6. Figure S4. Effects of tea polyphenols on composition of methanogens (genus level) in in vitro fermentation. S7. Figure S5. Effects of tea polyphenols on beta diversity of protozoa in in vitro fermentation. S8. Figure S6. Effects of tea polyphenols on alpha diversity of protozoa in in vitro fermentation. S9. Figure S7. Effects of tea polyphenols on the composition of protozoa at phylum level in in vitro fermentation. S10. Figure S8. Effects of tea polyphenols on the composition of protozoa at genus level in in vitro fermentation. S11. Figure S9. Biological process of differentially expressed proteins in rumen epithelium cells. S12. Figure S10. Cellular component of differentially expressed proteins in rumen epithelium cells. S13. Figure S11. Parallel reaction monitor mass spectrum.
