# Supplementary material for: Tea Polyphenols Inhibit Methanogenesis and Improve Rumen Epithelial Transport in Dairy Cows
Source: Animals (Basel). 2024 Sep 4;14(17):2569. doi: 10.3390/ani14172569 (PMC11394105; doi:10.3390/ani14172569)
Supplement: Supplementary file 1 [file animals-14-02569-s001.zip › animals-3171335-supplementary.pdf]

S1 Composition of artificial saliva

Table S1 Composition of artificial saliva

Category	Composition and dosage
Micromineral solution（A）	$CaCl_2 \cdot 2H_2O$ 13.2 g
	$MnCl_2 \cdot 4H_2O$ 10.0 g
	$CoCl_2 \cdot 6H_2O$ 1.0 g
	$FeCl_3 \cdot 6H_2O$ 8.0 g
	Dissolve in distilled water to a volume of 100 mL
Buffer solution（B）	$NaHCO_3$ 35.0 g
	NH_4HCO_3 4.0 g
	Dissolve in distilled water to a constant volume of 1000 mL and prepare as needed.
Macromineral solution（C）	$Na_2HPO_4 \cdot 12H_2O$ 9.45 g
	KH_2PO_4 6.2 g
	$MgSO_4 \cdot 7H_2O$ 0.6 g
	Dissolve in distilled water to a volume of 1000 mL
Resazurin aqueous（D）	Resazurin 100 mg
	Dissolve in distilled water to a volume of 100 mL
Reducing solution（E）	1M NaOH 2.0 mL
	$Na_2S \cdot 9H_2O$ 336 mg
	Add 47.5 mL of distilled water, configure on the day of cultivation

S2 Ratio of artificial saliva

Table S2 Ratio of artificial saliva

Category	Volume (mL)
Micromineral solution (A)	0.12
Buffer solution (B)	237.00
Macromineral solution (C)	237.00
Resazurin aqueous (D)	1.22
Reducing solution (E)	49.50
Distilled water	474.00

S3 Effects of tea polyphenols on alpha diversity of methanogens in *in vitro* fermentation

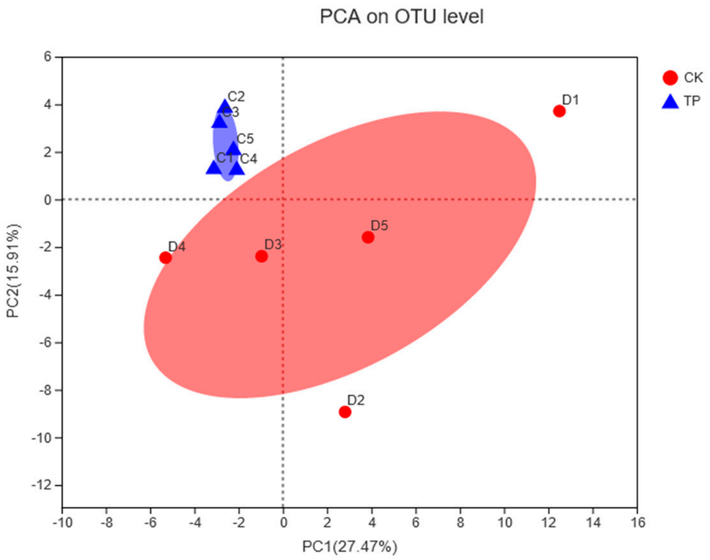

Figure S1 Effects of tea polyphenols on beta diversity of methanogens in *in vitro* fermentation. CK: control group; TP: tea polyphenols.

S4 Effects of tea polyphenols on alpha diversity of methanogens in *in vitro* fermentation

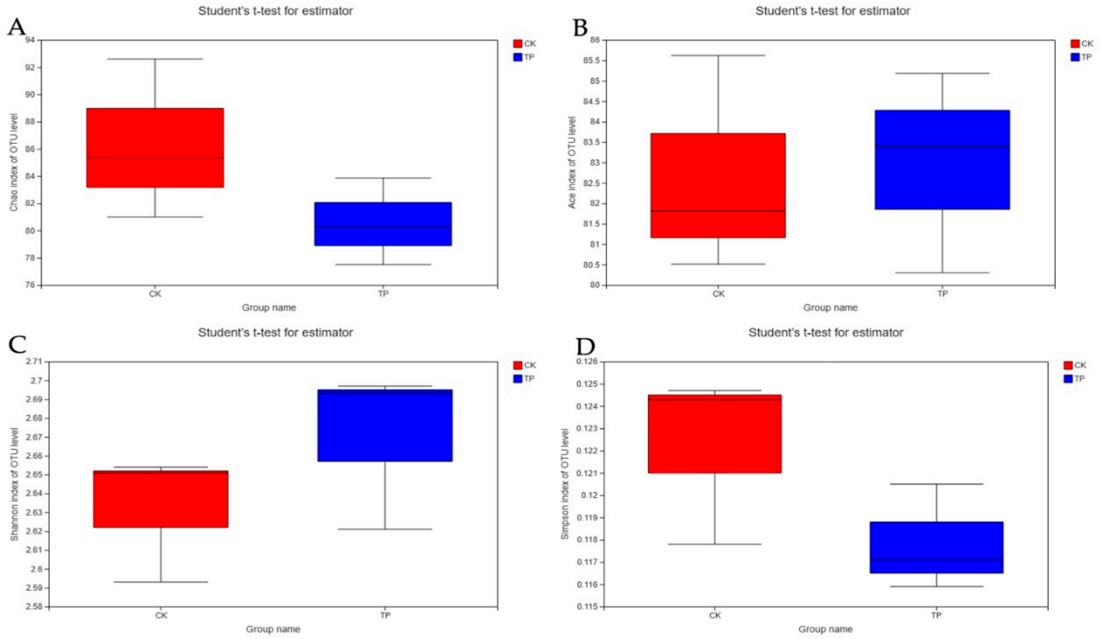

Figure S2 Effects of tea polyphenols on beta diversity of methanogens in *in vitro* fermentation. CK: control group; TP: tea polyphenols.

S5 Effects of tea polyphenols on the composition of methanogens (phylum level) in *in vitro* fermentation

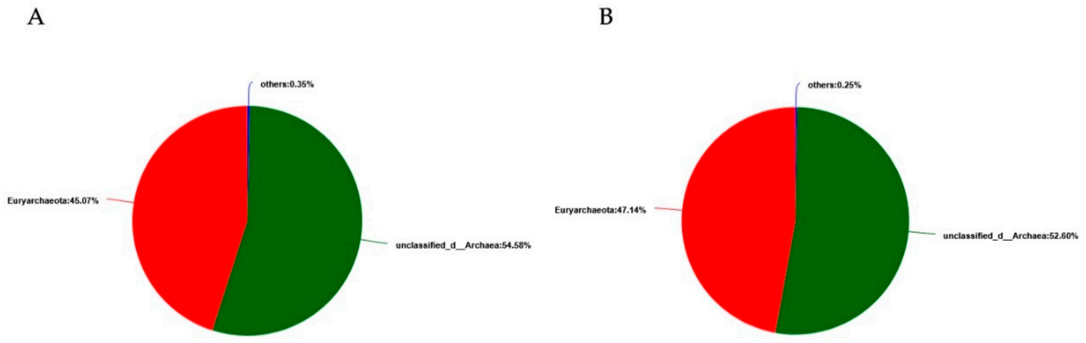

Figure S3 Effects of tea polyphenols on composition of methanogens (phylum level) in *in vitro* fermentation. CK: control group; TP: tea polyphenols.

S6 Effects of tea polyphenols on composition of methanogens (genus level) in *in vitro* fermentation

A

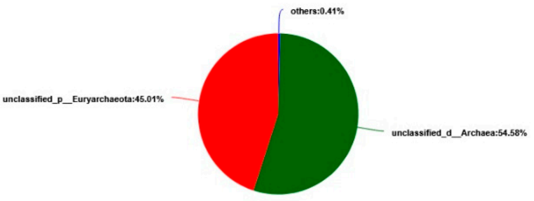

B

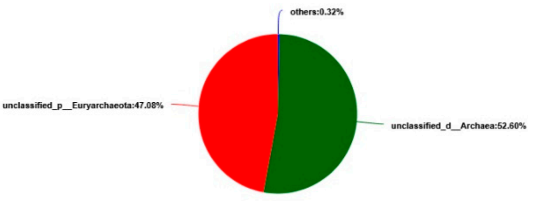

Figure S4 Effects of tea polyphenols on composition of methanogens (genus level) in *in vitro* fermentation. A: control group; B: tea polyphenols.

S7 Effects of tea polyphenols on beta diversity of protozoa in *in vitro* fermentation

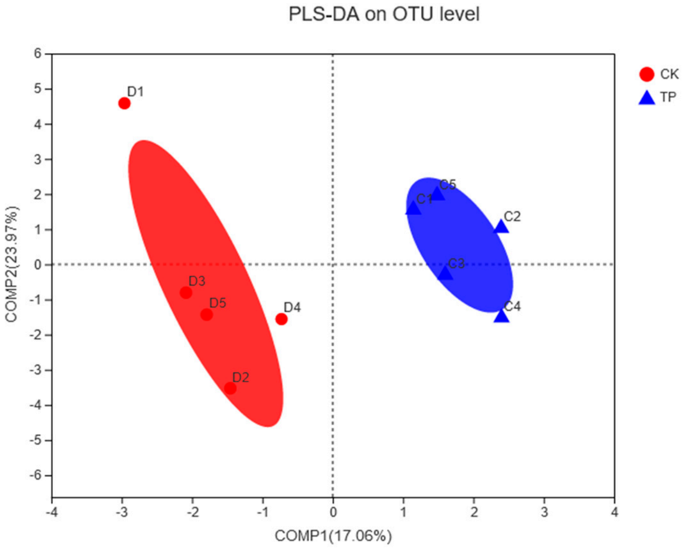

Figure S5 Effects of tea polyphenols on beta diversity of protozoa in *in vitro* fermentation. CK: control group; TP: tea polyphenols.

S8 Effects of tea polyphenols on alpha diversity of protozoa in *in vitro* fermentation

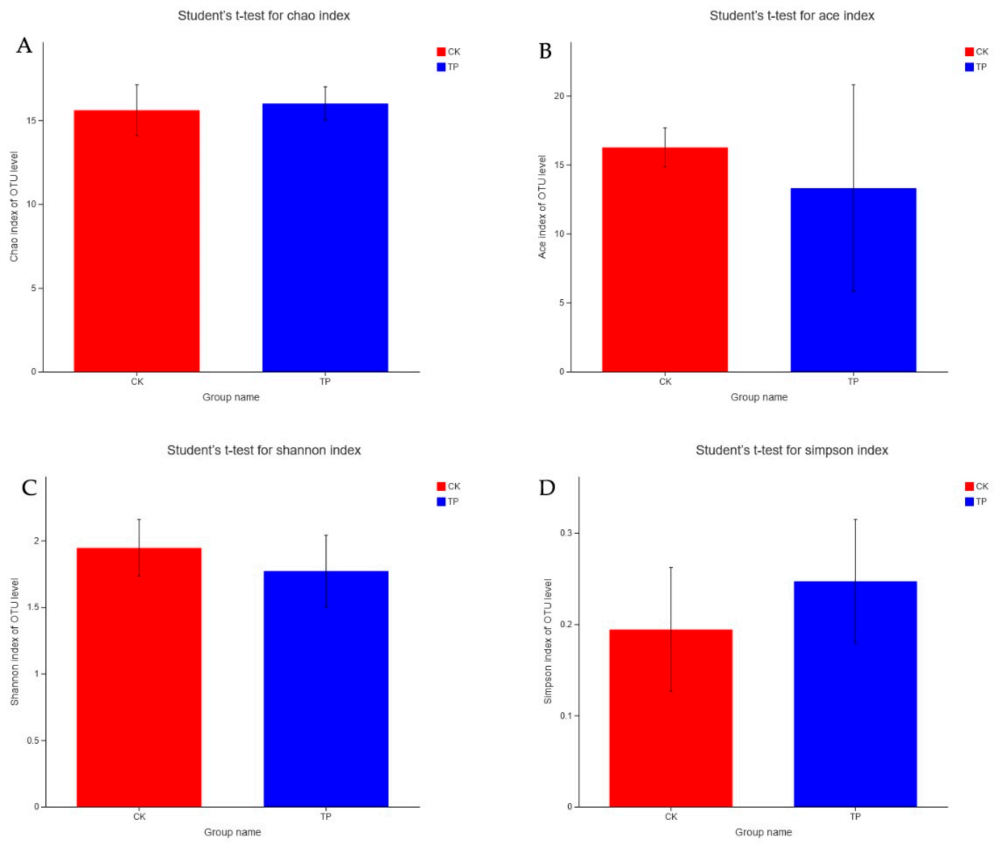

Figure S6 Effects of tea polyphenols on alpha diversity of protozoa in *in vitro* fermentation. CK: control group; TP: tea polyphenols.

S9 Effects of tea polyphenols on the composition of protozoa at phylum level in *in vitro* fermentation

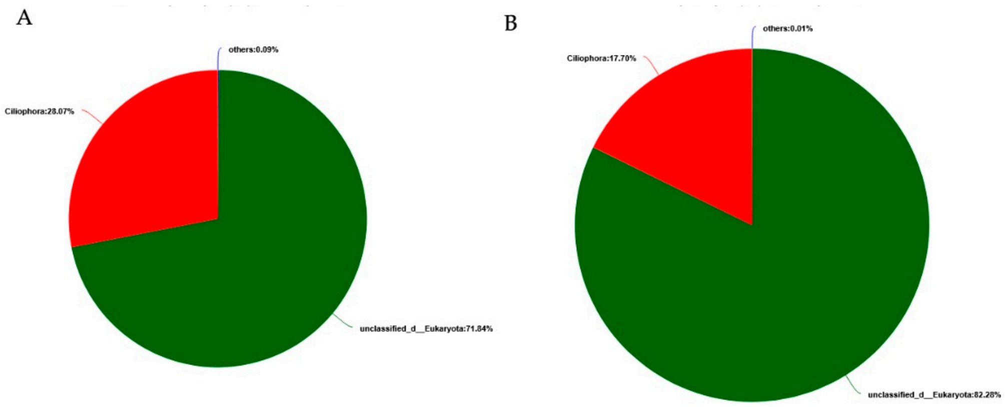

Figure S7 Effects of tea polyphenols on composition of protozoa at phylum level in *in vitro* fermentation. A: control group; B: tea polyphenols.

S10 Effects of tea polyphenols on the composition of protozoa at genus level in *in vitro* fermentation

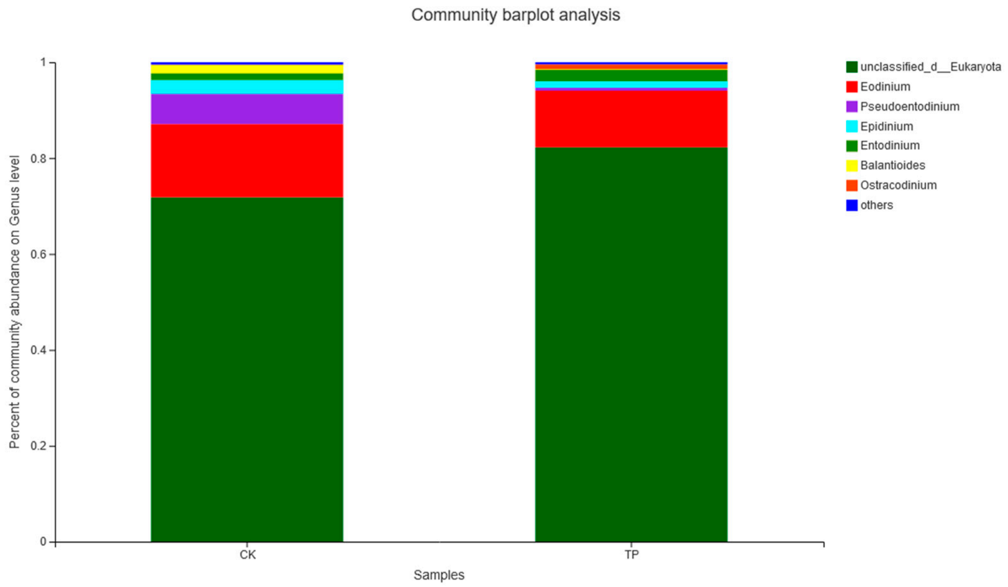

Figure S8 Effects of tea polyphenols on composition of protozoa at genus level in *in vitro* fermentation. CK: control group; TP: tea polyphenols.

S11 Biological process of differentially expressed proteins in rumen epithelium cells

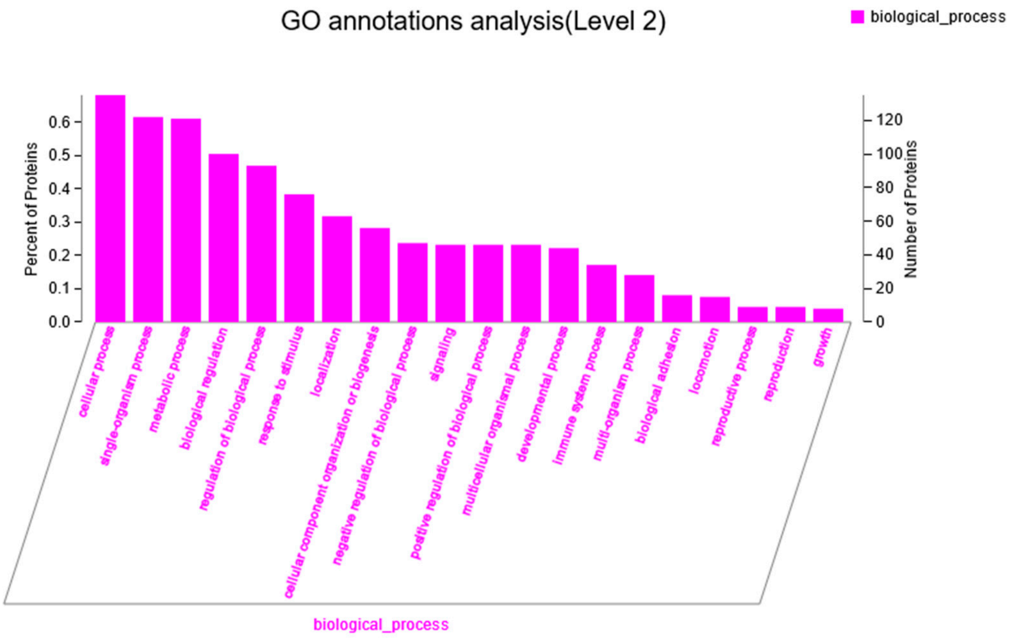

Figure S9 Biological Process of differentially expressed proteins in rumen epithelium cells

S12 Cellular component of differentially expressed proteins in rumen epithelium cells

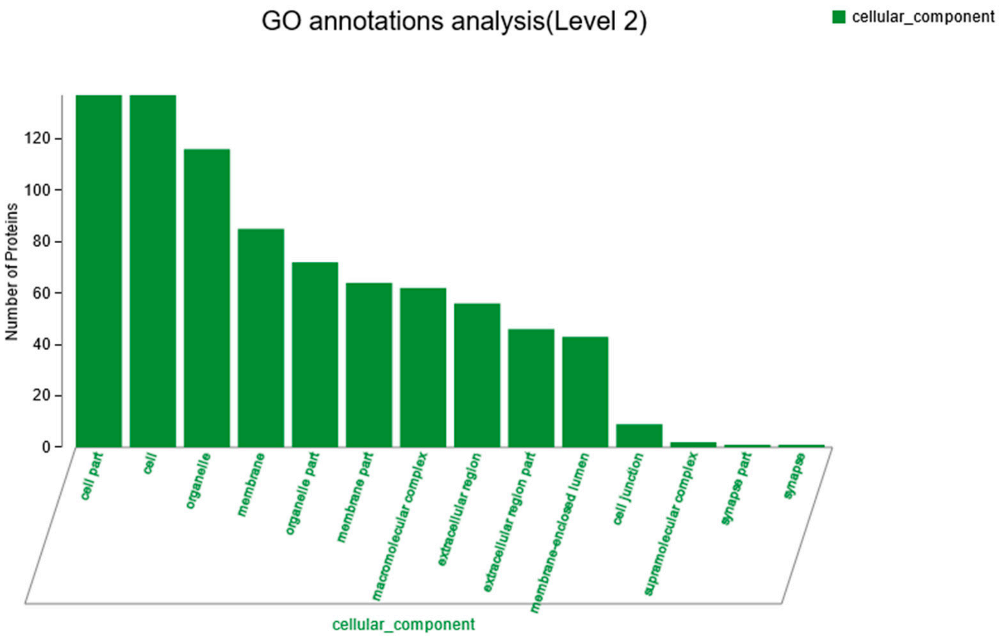

Figure S10 Cellular Component of differentially expressed proteins in rumen epithelium cells

S13 Parallel reaction monitor mass spectrum

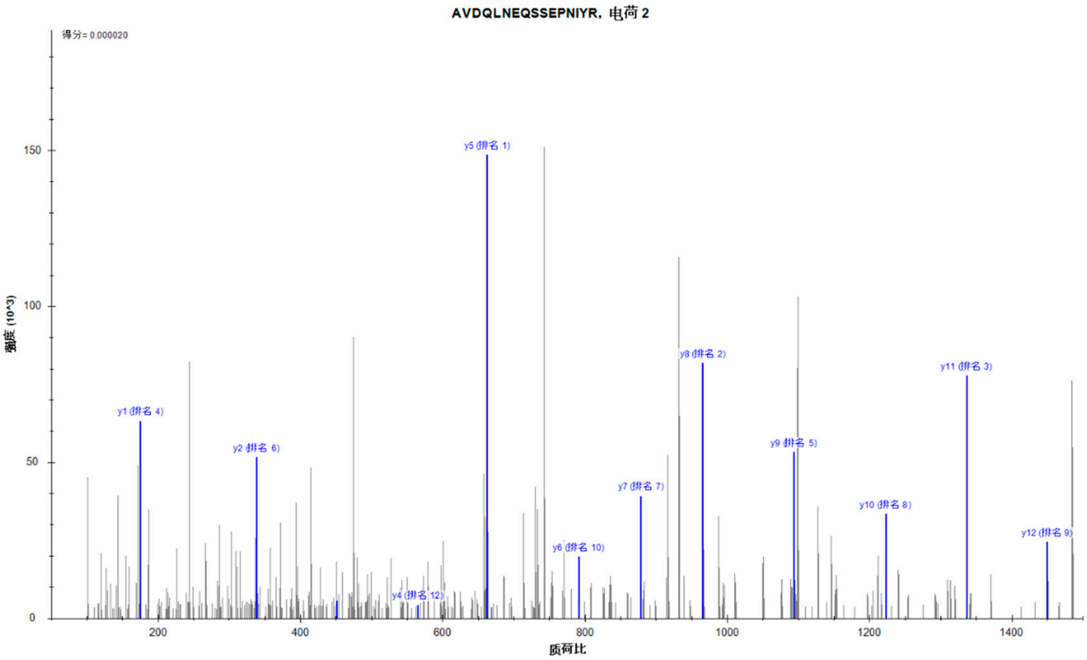

Figure S11 Parallel reaction monitor mass spectrum
